# Toxicity profiles of immune checkpoint inhibitors in nervous system cancer: a comprehensive disproportionality analysis using FDA adverse event reporting system

**DOI:** 10.1007/s10238-024-01403-2

**Published:** 2024-09-09

**Authors:** Rongrong Liu, Hui Zhao, Zenghong Lu, Lingshuai Zeng, Huaqiu Shi, Longqiu Wu, Jing Wang, Fangjun Zhong, Chuanjian Liu, Yu Zhang, Zhengang Qiu

**Affiliations:** 1 Department of Neurology, The First Affiliated Hospital of Gannan Medical University, Ganzhou, Jiangxi China; 2 Department of Sleep Medicine, Ganzhou People’s Hospital, Ganzhou, Jiangxi China; 3 Department of Oncology, The First Affiliated Hospital of Gannan Medical University, Ganzhou, Jiangxi China; 4 Major of Rehabilitation, Faculty of Medicine, Jinggangshan University, Ji’an, Jiangxi China

**Keywords:** FDA adverse event reporting system, Immune checkpoint inhibitors, Nervous system cancer, Immune-related adverse events, Real-world data

## Abstract

Background: Immune-related adverse events (irAEs) always occur during treatment with immune checkpoint inhibitors (ICIs). Patients with nervous system cancer (NSC) may gain clinical benefit from ICIs, but irAEs in NSC patients are rarely examined. Therefore, our study systematically summarized reports of irAEs in NSC. Methods: We obtained information from the FDA adverse event reporting system from the first quarter (Q1) of 2013 to the fourth quarter (Q4) of 2022. We examined use of a combination of ICIs and chemotherapy (ICI_Chemo) or chemotherapy only (ICI_Chemo) for patients with NSC. Multiple disproportionality analyses were applied to assess irAEs. Multiomics data from the gene expression omnibus (GEO) database were analyzed to explore potential molecular mechanisms associated with irAEs in NSC patients. Results: Fourteen irAEs were identified in 8,357 NSC patients after removing duplicates; the top five events were seizure, confused state, encephalopathy, muscular weakness and gait disturbance. Older patients were more likely to develop irAEs than were younger patients. From the start of ICIs_Chemo to irAE occurrence, there was a significant difference in the time to onset of irAEs between age groups. irAEs may occur via mechanisms involving the inflammatory response, secretion of inflammatory mediators, and aberrant activation of pathologic pathways. Conclusions: This study helps to characterize irAEs in NSC patients treated with ICIs. We combined GEO database analysis to explore the potential molecular mechanisms of irAEs. The results of this study provide a basis for improving the toxic effects of ICIs in NSC patients.

**Supplementary Information:**

The online version contains supplementary material available at 10.1007/s10238-024-01403-2.

## Introduction

Surgical, radiotherapy and chemotherapy treatments for nervous system cancer (NSC) are evolving, but NSC recurrence seems inevitable [1]. The recent development of immune checkpoint inhibitors (ICIs) [mainly including anti-programmed death-1 (PD-1)/programmed death-ligand 1 (PD-L1) and anti-cytotoxic T-lymphocyte-associated protein 4 (CTLA-4)] has revolutionized treatment of all cancer types, resulting in durable responses and acceptable toxicities [2]. Although ICIs have been less effective in NSC, some studies have shown that ICIs can enhance T-cell responses in the tumor microenvironment (TME) and prolong the mean survival time of these patients [2–4]. Overall, the composition of the TME is a key determinant of tumor-immune interactions and is an important factor influencing the efficacy of tumor immunotherapy [5].

NSCs are highly heterogeneous, with a highly inhibitory TME and low immunogenicity, which limit an effective immune response and render immunotherapy for these patients extremely challenging [6]. NSCs present distinct challenges for immunotherapy compared to other solid tumors. The central nervous system (CNS) has long been considered an immune-privileged site due to the blood–brain barrier (BBB) and the lack of conventional lymphatic drainage, which can hinder the infiltration and activity of immune cells [6]. Moreover, the unique immunosuppressive TME in NSCs, characterized by the presence of immunosuppressive cells like regulatory T cells (Tregs) and myeloid-derived suppressor cells (MDSCs), as well as immunosuppressive cytokines like TGF-*β* and IL-10, can dampen the efficacy of immunotherapies [5, 6]. Additionally, the heterogeneity and complex molecular subtypes of NSCs, pose challenges in identifying suitable patient populations for immunotherapy, but in most cases, these immune interactions are effectively suppressed by the NSCs [3, 4].

Inhibition of the PD-1/PD-L1 immunoregulatory axis helps to enhance the proliferation, activation, and infiltration of cytotoxic lymphocytes (CTLs) and reduce expression of regulatory T cells (Tregs), thereby restoring immune function and improving the prognosis of NSC patients [4, 7]. A phase II clinical study found that among patients with surgically resectable recurrent glioblastoma (GBM), those who underwent preoperative PD-1 monoclonal antibody treatment had a significantly longer overall survival (OS) time than patients who underwent postoperative adjuvant therapy, with the TME of GBM being significantly activated [4]. Another clinical trial (NCT03233152) confirmed the favorable efficacy of anti-PD-1 antibodies in combination with CTLA-4 for treatment of GBM [8]. The combination of a monoclonal antibody and radiation therapy to block PD-L1 on glioma cells also resulted in significantly longer survival times in preclinical mouse models [9]. Therefore, ICIs may become a novel treatment approach for NSC patients [2–4, 10].

ICIs have improved the survival of cancer patients. However, as ICIs are being more widely used in clinical practice, a key challenge has arisen, namely, uncontrolled side effects on the immune system, which may lead to immune-related adverse events (irAEs). According to the FDA adverse event reportingsystem (FAERS), a total of 149,303 cases of adverse events have been reported [11]. Unlike chemotherapy and targeted therapies, most of the irAEs caused by ICIs are caused by overimmunization against normal organs; furthermore, the severity of irAEs, the time of onset (TTO), and the organs involved are often unpredictable [12]. irAEs can lead to delayed or interrupted treatment and, in rare cases, may be life-threatening [13, 14]. Given these unique challenges, it is crucial to comprehensively evaluate the safety profiles and irAEs associated with ICIs specifically in NSC patients. While, the toxicity profiles of ICIs have been extensively studied in other cancer types, a focused analysis in NSCs is lacking. Our study aims to address this gap by leveraging real-world data from the FDA adverse event reporting system (FAERS) to provide a comprehensive assessment of irAEs in NSC patients treated with ICIs. This focused analysis will help inform clinical decision-making and management of irAEs in this specific patient population, ultimately optimizing the risk–benefit ratio of immunotherapy in NSCs. Hence, in this study, we summarized irAEs in real-world NSC patients. Additionally, we explored the molecular mechanisms associated with irAEs in NSC patients in conjunction with multiomics data from the Gene Expression Omnibus (GEO) database to improve clinicians' knowledge about irAEs in NSC patients and to avoid a decrease in ICI therapeutic efficacy due to adverse drug reactions of NSC.

## Methods

### Data sources and preprocessing

We downloaded the FAERS data files from the FDA website (https://fis.fda.gov/extensions/FPD-QDE-FAERS/FPD-QDE-FAERS.html) for the period of Q1-2013 to Q4-2022. The downloaded files included six datasets: patient demographic and administrative information (DEMO), drug information (DRUG), adverse event information (REAC), patient outcomes (OUTC), drug therapy start and end dates (THER), and indications for use/diagnosis (INDI). The data preprocessing steps included removing duplicate records, standardizing drug names, and mapping the adverse events to the preferred terms (PTs) in the Medical Dictionary for Drug Regulation Activities (MedDRA) 0.14. Duplicate reports in the raw data were excluded according to the literature, and erroneous events were removed according to the officially provided Deleted file. Only primary suspect (PS) drugs were selected as ICIs or chemotherapy (Supplementary Table 1), and the indications for medication were 'Nervous system neoplasms malignant and unspecified NEC' [high-level group term (HLGT) from the MedDRA. We extracted target drug-related adverse event reports coded according to the PT of MedDRA. Use of ICIs in combination with chemotherapy was categorized as ICI_Chemo; use of chemotherapy only was categorized as Only_Chemo. After removing duplicates, adverse event reports from 8,357 patients were ultimately included in this study.

### Signal judgment of irAEs

We used the proportional reporting ratio (PRR), information component (IC), and reporting odds ratio (ROR) for calculation of adverse event signals. To improve the accuracy of signal identification, the presence of irAEs was defined as a ≥ 3, ROR025 ≥ 1 [15], IC025 > 1 and PRR > 2, X^2^_yates_ ≥ 4 [16].

### Exploration of the molecular mechanisms of irAEs

We downloaded mRNA expression data for 20 NSCs from the GEO database (https://www.ncbi.nlm.nih.gov/geo/query/acc.cgi) (GSE49710, GSE85047, GSE3446, GSE120559, GSE120568, GSE4271 GSE2727, GSE23869, GSE33331, GSE19728, GSE13041, GSE58399, GSE7696, GSE5107, GSE134783, GSE107850, GSE43378, GSE43289, GSE2817 and GSE43113), including cancer types such as neuroblastoma, astrocytoma, glioblastoma (GBM) and glioma (Supplementary Table 2). Next, the ‘GSVA’ package [17] and single-sample gene set enrichment analysis (ssGSEA) algorithms were applied to calculate pathway enrichment scores for each cancer patient. Additionally, we collected immune-related gene sets from a previous study [18, 19]. The median ssGSEA score for each cancer type was calculated based on the ssGSEA score for each cancer patient [20]. Similarly, the median immune-related gene expression for each cancer type was calculated based on the immune-related gene expression of each cancer patient. We used the ‘caret’ package to construct predicted irAE RORs in bivariate linear regression models based on ssGSEA scores or immune-related gene expression for different cancer types. Immune-related genes that correlated significantly with the irAE RORs were then used as input files for gene set enrichment analysis (GSEA) for subsequent analysis.

### Statistical analysis

Univariate and multivariable logistic regression analyses were performed to explore whether the occurrence of irAEs, sex, and age had an impact on the prognosis of NSC patients. Similarly, univariate and multivariable logistic regression models were used to explore the effects of sex and age on the occurrence of irAEs in NSC patients. Heatmaps, histograms and box plots were then generated based on the ‘ggplot2’ package [21]. GSEA was implemented through the ‘clusterprofiler’ package [22] in R. We used Spearman's correlation analysis [23] to assess correlations between (i) the predicted irAE RORs and irAE RORs, (ii) the ssGSEA score and irAE RORs and (iii) expression of immune-related genes and the irAE RORs. The log likelihood ratio test was applied to assess the fit of bivariate linear regression models. The definition of age groupings of Lin et al [24]. was used in this study. Data analysis and picture visualization for this study were performed in R software (V4.1.1). A two-sided p value of less than 0.05 was considered significantly different.

## Results

### irAEs in real-world NSC patients

**Fig. 1:**
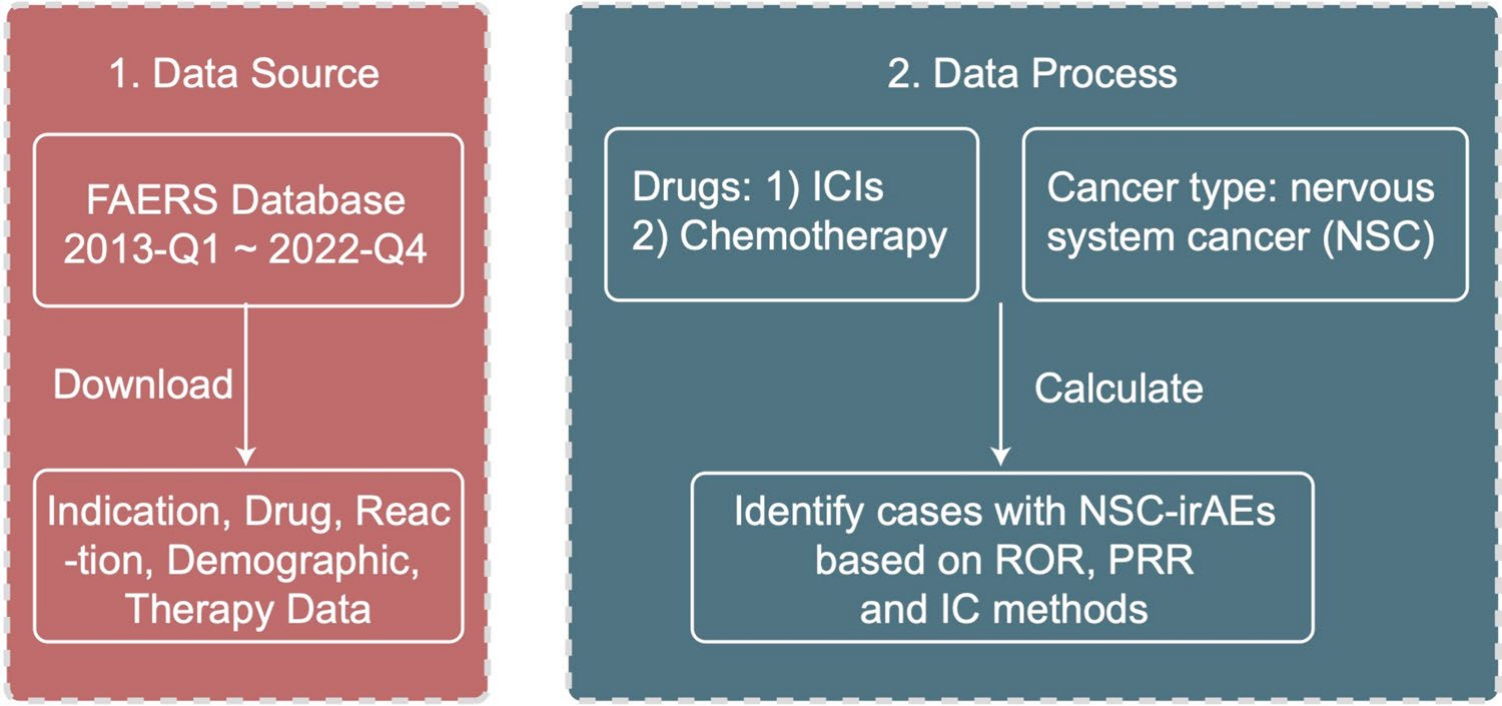
Flow diagram of the definition of irAEs in NSC

**Fig. 2:**
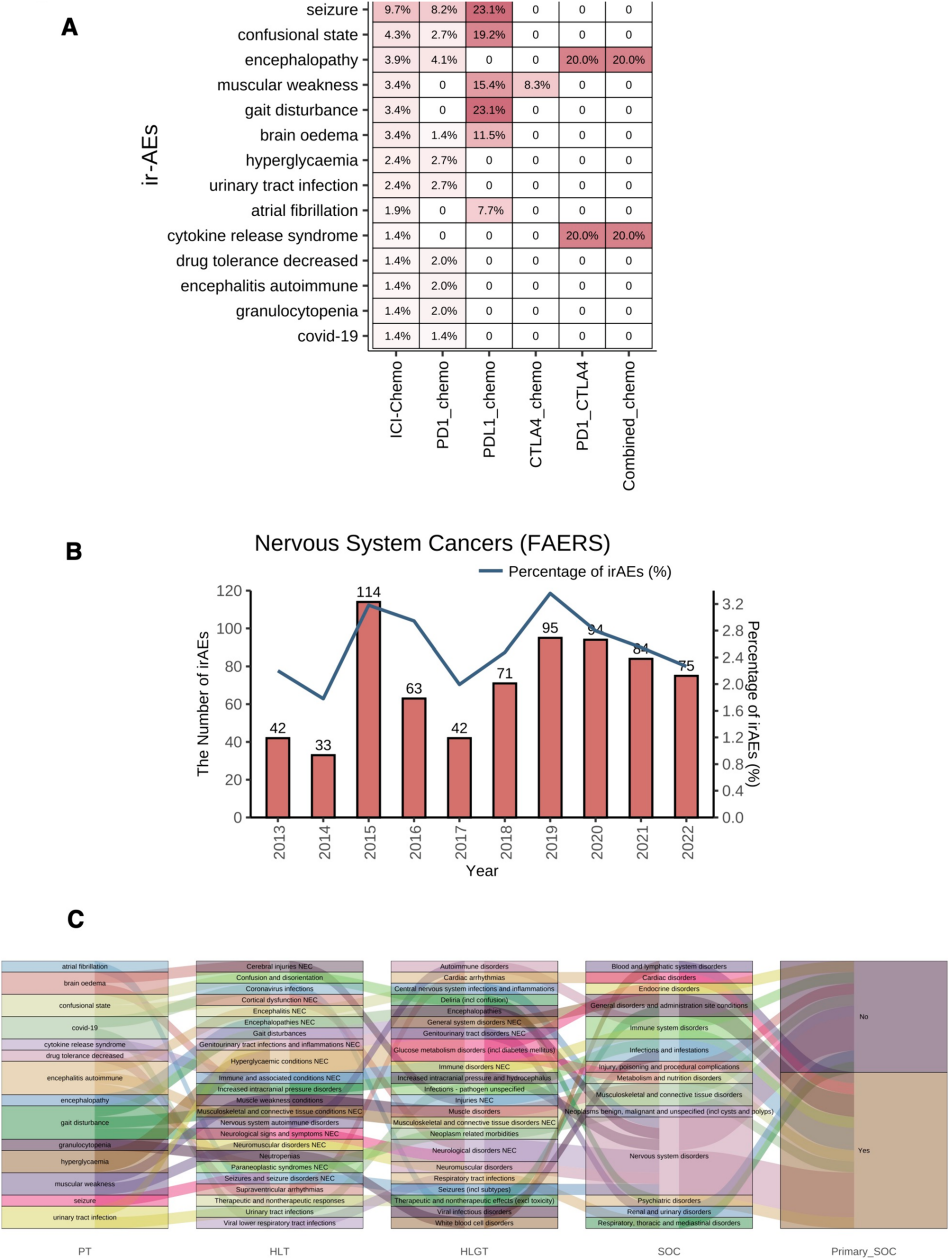
irAEs of NSC in real-world data. **A** The heatmap depicts the 14 irAEs based on the disproportionality analysis in FAERS. **B** The bar plot depicts the case number or percentages of irAEs between 2013 and 2022. **C** The Sankey plot depicts the high-level term (HLT), high-level group terms (HLGT) and system organ class (SOC) of the 14 irAEs based on MedDRA

Based on the adverse reaction reports of NSC patients who received ICI_Chemo or Only_Chemo after removing duplicate cases, we combined three adverse reaction signal detection algorithms (PRR, ROR, and IC) to compute and judge irAEs (Fig. 1). In total, we identified 14 irAEs in NSC patients (Fig. 2A). The irAEs that occurred in NSC patients using ICI_Chemo in descending order of frequency were seizure, confused state, encephalopathy, muscular weakness, gait disturbance, brain edema, hyperglycemia, urinary tract infection, atrial fibrillation, cytokine release syndrome, decreased drug tolerance, autoimmune encephalitis, granulocytopenia and COVID-19 (Supplementary Table 3). Between 2013 and 2022, the frequency of irAEs in NSC patients ranged from 2.0 to 3.4%, with a case number ranging from 42 to 114 (Fig. 2B). Figure 2C shows 14 irAEs categorized at the high-level term (HLT), HLGT and system organ class (SOC) level categorization.

### Relationship between irAEs and medication regimen, sex, and age

**Fig. 3:**
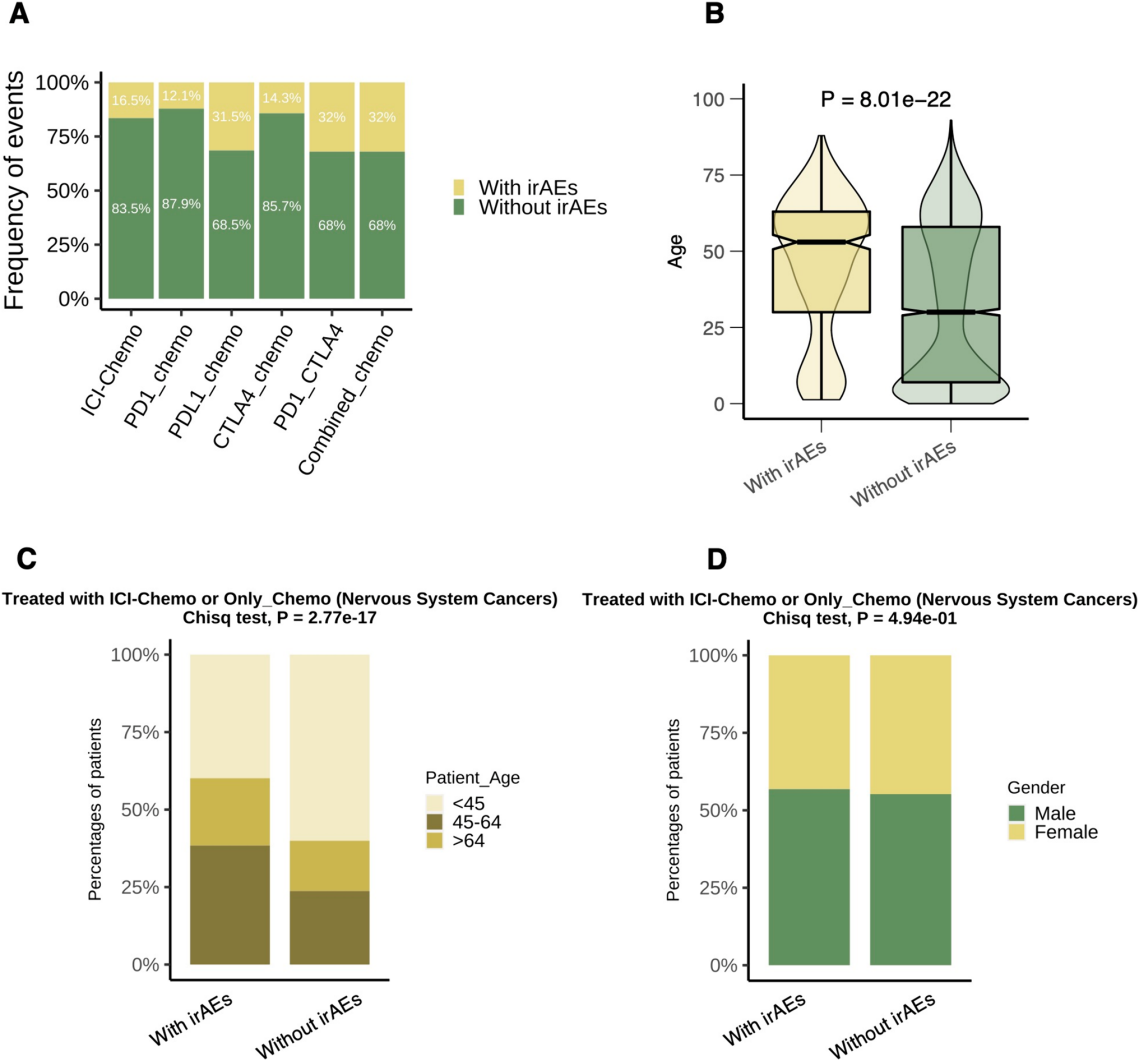
Analysis of the association between irAEs and clinical characteristics. **A** The bar plot depicts the percentage of irAEs among different drug subgroups. **B** The violin boxplot depicts differences in age between groups with and without irAEs. **C** Differences in the proportion of individuals in different age groups (< 45, 45–64, > 64) between patients with and without irAEs. **D** Differences in the percentage of individuals of the different sexes between patients with and without irAEs

We found that the proportions of irAEs varied among ICI regimens (Fig. 3A), with the highest proportions occurring in combination regimens (PD1_CTLA4_Chemo: 32.0%; Combined-ICI_Chemo: 32.0%) and the lowest in monotherapy regimens (PD1_Chemo: 12.1%, CTLA4_Chemo: 14.3%). Unexpectedly, irAEs occurred in 31.5% of CTLA4_Chemo regimens. Regarding age, we found a significantly higher proportion of patients > 64 years of age than NSC patients without irAEs among NSC patients with irAEs (Fig. 3C, *P* < 0.05). There was no significant difference in the proportion of males and females in NSC patients with irAEs compared with those without irAEs (Fig. 3C, *P* > 0.05).

### Relationships between medication regimens, sex, age, and time to onset and status of irAEs

**Fig. 4:**
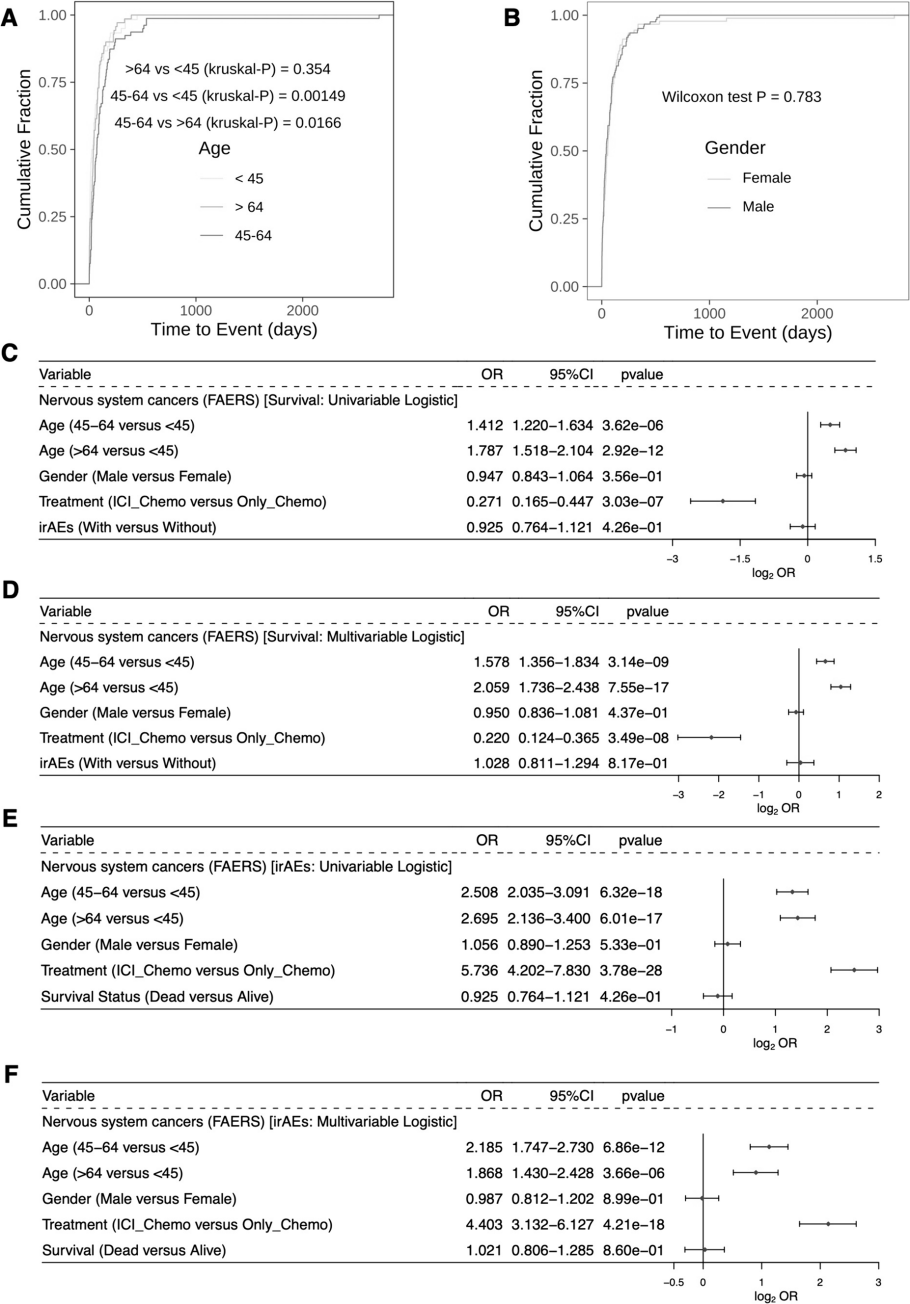
Analysis of the association between irAEs and prognosis. Cumulative distribution function of irAEs by time to event grouped by age (**A**) and sex (**B**). Univariable (**C**) and multivariable (**D**) logistic regression models of clinical characteristics and irAEs with the prognosis of cancer patients. Univariable (**E**) and multivariable (**F**) logistic regression models of the clinical characteristics and prognosis of irAEs in cancer patients

With respect to age, we found a significant difference in the TTO of irAEs in NSC patients between two age subgroups (45–64 vs. > 64, *P* < 0.05; 45–64 vs. < 45, *P* < 0.05, Fig. 4A). In contrast, there was no statistically significant difference between male and female NSC patients in the TTO of irAEs (Fig. 4B, *P* > 0.05). The univariable logistic regression model showed that older age and receiving only chemotherapy regimens were risk factors for survival in NSC patients (Fig. 4C, OR > 1, *P* < 0.05). According to the multivariable logistic regression model, older age and receiving only chemotherapy regimens may serve as independent predictive factors for survival in NSC patients (Fig. 4D, OR > 1, *P* < 0.05). Similarly, we found that older NSC patients who received ICI_Chemo were more likely to develop irAEs, thus indicating that ICI_Chemo is an independent predictor of irAEs (Fig. 4E, F, OR > 1, *P* < 0.05).

### Analysis of irAEs in the GEO database

**Fig. 5:**
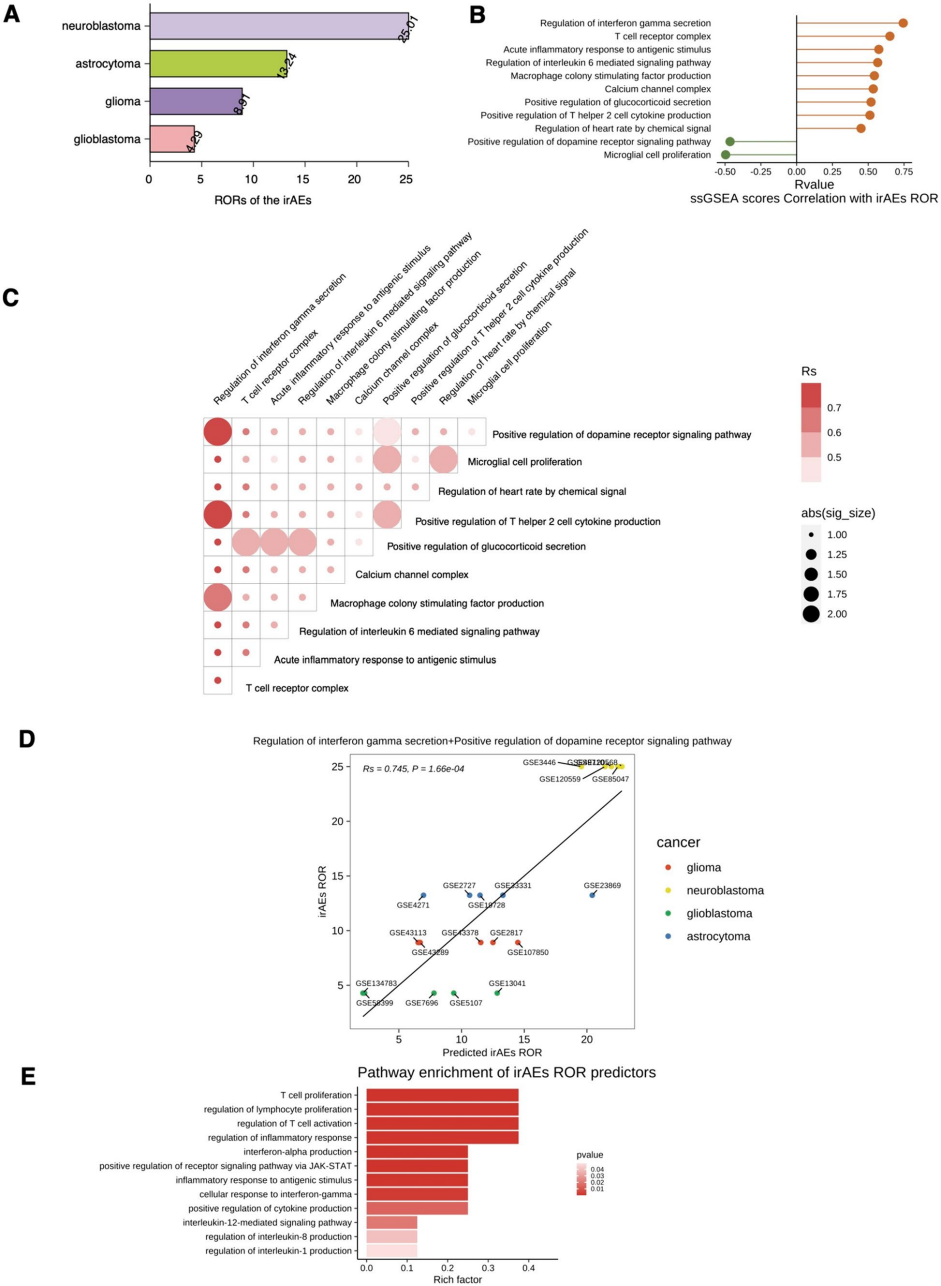
Analysis of the association between irAEs and related factors. **A** irAEs ROR across different cancer types. **B** Spearman correlation analysis between the irAE RORs and ssGSEA score of the 11 signaling pathways for positive correlation (orange lollipop) and negative correlation (green lollipop). **C** Comparison of the performance of bivariate models in predicting irAEs for all combinations of fourteen significantly correlating variables. Spearman’s correlation coefficient (Rs) was calculated between predicted and observed irAE RORs. The shade of the circle indicates the Rs, and the size indicates the significance of the log-likelihood ratio test. **D** Combined effect of regulation of interferon gamma secretion and positive regulation of the dopamine receptor signaling pathway in the bivariate model (Spearman correlation, 0.808, *P* < 0.05). **E** Pathway enrichment of significantly immune-related genes correlating with the irAE

The highest ranked irAE RORs was for neuroblastoma (Fig. 5A, Supplementary Table 4, ROR = 25.01), followed by astrocytoma (ROR = 13.24), glioma (ROR = 8.91) and GBM (ROR = 4.29). We found that regulation of interferon gamma secretion, T-cell receptor complex, acute inflammatory response to antigenic stimulus, regulation of interleukin six-mediated signaling pathway, macrophage colony stimulating factor production, calcium channel complex, positive regulation of glucocorticoid secretion, positive regulation of T helper 2 cell cytokine production and regulation of heart rate by chemical signal correlated positively with irAEs (Rs > 0, *P* < 0.05, Fig. 5B). In contrast, microglial cell proliferation and positive regulation of the dopamine receptor signaling pathway were negatively associated with irAEs (Rs < 0, *P* < 0.05, Fig. 5B). To identify more predictive models, we combined the above pathways significantly associated with irAEs (Supplementary Fig. 1) and assessed their predictive power using bivariate linear regression models and log-likelihood ratio tests (Supplementary Table 5, Supplementary Figs. 2 and 5B). The combined model of regulation of interferon gamma secretion and positive regulation of the dopamine receptor signaling pathway achieved significant improvement in model predictive power compared to a single model (Supplementary Fig. 1). The combination of regulation of interferon gamma secretion and positive regulation of the dopamine receptor signaling pathway also led to significant improvement in the predictive power of the model compared to a single factor (log-likelihood ratio test *P* < 0.05, Fig. 5C). Moreover, the combined model of regulation of interferon gamma secretion and positive regulation of the dopamine receptor signaling pathway maximized prediction of irAEs (Rs = 0.745, *P* < 0.05, Fig. 5D). Immune genes significantly associated with irAEs (Supplementary Table 6) were enriched for interferon-alpha production, regulation of lymphocyte proliferation, positive regulation of receptor signaling pathway via JAK-STAT, regulation of T-cell activation, inflammatory response to antigenic stimulus, regulation of inflammatory response, cellular response to interferon-gamma, positive regulation of cytokine production, interleukin-12-mediated signaling pathway, regulation of interleukin-8 production and regulation of interleukin-1 production (Fig. 5E, Supplementary Table 7, *P* < 0.05).

## Discussion

Currently, the standard treatment for most NSCs remains surgical resection combined with radiation therapy and chemotherapy [25]. Despite the emergence of many therapeutic agents in recent decades, NSC patients often experience recurrence, which is largely due to the highly aggressive and heterogeneous nature of the cancer [26]. In recent years, ICIs, as a promising anticancer tool, have significantly improved the clinical prognosis of cancer patients alongside surgical, radiotherapeutic and chemotherapeutic approaches [10]. PD-1/PD-L1 and CTLA-4 inhibitors have been approved by the FDA and have achieved favorable efficacy in non-small cell lung cancer (NSCLC), melanoma and renal cell carcinoma (RCC) [27–30]. Given the long-lasting therapeutic efficacy of ICIs in other cancers and our increased knowledge of the immune function of NSC, there is some theoretical basis for using ICIs in NSC patients. As a result, clinical trials have been conducted to apply ICIs to a wide range of NSC patients [26]. Cloughesy et al. found that patients with relapsed GBM treated with neoadjuvant pembrolizumab + continuous postoperative adjuvant therapy had significantly longer OS than patients with recurrent GBM in the chemotherapy-only group; the former patients showed a greater tendency toward T-cell clonal expansions in the TME and had a higher prevalence of peripheral blood T cells with reduced levels of PD-1 expression on T cells [4]. The survival rates of brain cancer in mice treated with anti-CTLA-4 and anti-PD-1 monotherapy were 40% and 60%, respectively, whereas the survival rate was up to 78% when the two were combined [31]. Additionally, Reardon et al. [32] found that combination therapy (targeting CTLA-4 and PD-1) cured 75% of GBM mice and induced a cancer-specific memory effect, further inhibiting cancer recurrence. Nevertheless, immunotherapy-related adverse effects triggered by use of ICIs in NSC patients in the real-world have not yet been fully clarified. In this study, we systematically explored irAEs triggered by use of ICIs in NSC patients with the help of adverse reaction data from the real-world. In addition, expression data from the GEO database were used to analyze the molecular mechanisms potentially associated with the occurrence of irAEs after use of ICIs in NSCLC patients. We hope that these findings lead to more efficient and individualized therapeutic efficacy in NSC patients.

Seizures are the most common irAE, and the inflammatory response, inflammatory mediator secretion and aberrant activation of pathological pathways play integral roles in the development of seizures [33–36]. Reactive astrocytes and microglia have been found to produce proinflammatory cytokines in patients with seizures and lower the threshold for epileptogenesis [33, 34]. A significant increase in proinflammatory cytokines, including IFN-*γ*, IL-1*β*, IL-6, IL-10, IL-12, and TNF*α* [35], was observed after epileptogenesis, and levels of these inflammatory factors may serve as biomarkers for epilepsy [37]. Additionally, microglia play an important role in suppressing neuronal hyperactivity [38–40]. Studies suggest that microglia are beneficial in helping to restore dysfunctional neuronal structures after seizures [41]. Increased levels of intracellular Ca2 + may promote seizures, maintenance and propagation [42]. In this study, we found that regulation of interferon gamma secretion, regulation of the interleukin 6-mediated signaling pathway, acute inflammatory response to antigenic stimulus, and calcium channel complex correlated significantly and positively with the irAE RORs. In contrast, microglial cell proliferation correlated significantly and negatively. In addition, regulation of interleukin-1 production and interleukin-12-mediated signaling pathway were enriched among immune-relate genes significantly positive associated with irAEs.

Gait disturbance, hyperglycemia, and atrial fibrillation (AF) are also irAEs that frequently occur in patients with NSC. Alterations in intracellular Ca2 + may play an important role in the pathogenesis of AF [43], and abnormalities in Ca2 + metabolism may promote abnormalities in atrial potential conduction and affect the remodeling processes associated with AF [44–48]. Apoptosis of dopamine neurons in the substantia nigra compacta (SNc) leads to striatal dopamine deficiency, which affects the basal ganglia (BG) circuits involved in movement selection and planning, resulting in movement disorders such as tremor, bradykinesia, and difficulty in initiating voluntary movements [49]. Glucocorticoids cause hyperglycemia by disrupting various physiological metabolic mechanisms through increased insulin resistance and decreased insulin secretion [50, 51]. Overall, the effects of glucocorticoids on the liver, skeletal muscle and adipocytes lead to decreased insulin sensitivity [50]. In this study, we found that the calcium channel complex and positive regulation of glucocorticoid secretion correlated significantly and positively with the irAE RORs. In contrast, positive regulation of the dopamine receptor signaling pathway correlated significantly and negatively.

In addition to the above adverse reactions, cytokine release syndrome (CRS) and autoimmune encephalitis are also frequent irAEs in NSC patients. CRS is characterized by elevated levels of circulating cytokines [including INF-*γ*, TNF-*α*, IL-2, IL-6, IL-8, IL-10, and granulocyte–macrophage colony-stimulating factor (GM-CSF)], an acute systemic inflammatory response, and secondary organ dysfunction [52]. CRS usually occurs with CAR-T therapies but can also occur with ICIs [53–55]. According to analysis of the World Health Organization (WHO) global pharmacovigilance database, the incidence of ICI-induced CRS is approximately 0.07% [55]. Studies have shown that ICIs cause target-self immunotoxicity through immune system overactivation [52, 56]. In CRS, T cells, B cells, natural killer cells (NKs), macrophages, and endothelial cells release a variety of cytokines, among which IL-6 plays a central role [52, 56]. IL-6 activates downstream JAK-STAT3 signaling mainly by binding to IL-6R, inducing a strong proinflammatory signaling pathway [57]. Tocilizumab has become the mainstay of treatment of CRS due to its ability to block IL-6R and has been approved by the FDA [58]. Studies have shown autoimmune encephalitis to be a rare and potentially fatal adverse event following treatment with ICIs [59]. The incidence of ICI-associated encephalitis based on nivolumab, pembrolizumab, and atezolizumab dosing statistics was 0.2, 1, and 1%, respectively [60]. Given the extremely low frequency, the pathogenesis and mechanisms of autoimmune encephalitis are unclear and may include the following [61]. First, ICIs act as therapeutic antibodies that recognize target molecules (such as CTLA4 and PD-1/PD-L1) on intrinsic cells (endothelial cells, astrocytes, and neurons) within the nervous system, directly causing local damage through complement-dependent or cell-dependent cellular toxicity mechanisms [62]. Second, the immune response induced by ICIs may crossreact with CNS autoantigens [63]. In this study, we found that regulation of the interleukin six-mediated signaling pathway correlated significantly and positively with the irAE RORs. Additionally, signaling pathways such as T-cell proliferation or activation, JAK-STAT and inflammatory response were enriched among immune-related genes significantly positive correlated with irAEs.

COVID-19 is an irAE that has occurred in recent years after use of ICIs in patients with NSC. There are two main reasons for the impact of ICI treatment on COVID-19. The first is the potential overlap between two types of lung injury: use of ICIs and the possible pulmonary toxicity of COVID-19 pneumonia. The incidence of ICI-associated pneumonitis in anti-PD-1/PD-L1 monotherapy and anti-CTLA-4/anti-PD-1 combination therapy is reported to be 2.5–5% and 7–10% [64], respectively. These fatal immune-related adverse events account for 35% [65] of treatment-related deaths. A second concern is potential synergy between ICI-associated pneumonia and COVID-19 pathogenesis, both of which are associated with immune hyperactivation [66–68].

Our comprehensive analysis of real-world data has revealed the most common and clinically significant irAEs that occur in patients with nervous system cancers treated with ICIs, which include seizures, gait disturbance, hyperglycemia, AF, and CRS. Integrating data from the GEO database, we have also elucidated potential molecular pathways underlying the development of these irAEs, such as aberrant regulation of inflammatory cytokines, calcium signaling, and dopamine receptor signaling. These results provide a foundation for clinicians to proactively monitor for these critical irAEs in nervous system cancer patients receiving ICIs and to develop rational strategies for optimal patient selection and supportive care. For example, patients with pre-existing seizure disorders or neuroinflammatory conditions may not be ideal candidates for ICI therapy. For those treated with ICIs, prophylactic use of anti-epileptic drugs, calcium channel blockers, and agents targeting IL-6 and other key inflammatory mediators could potentially reduce the incidence and severity of irAEs. Ultimately, prospective studies are needed to validate the predictive utility of the molecular signatures identified here and to test the efficacy of mechanism-based interventions to improve the safety of ICI therapy in patients with nervous system cancers. By integrating real-world evidence with multiomics data, this work provides a promising roadmap for personalizing immune checkpoint blockade and irAE management in this challenging clinical context.

This study has several limitations that should be considered when interpreting the results. First, the number of identified irAEs in patients with nervous system tumors may be relatively low, given the complexity and variability of immune responses in oncology patients. The limited number of identified irAEs may not provide a comprehensive overview of the full spectrum of adverse events associated with immune checkpoint inhibitor therapy in this patient population. Future studies should consider exploring additional data sources and including a larger number of adverse events to gain a more complete understanding of the side effects of immunotherapy in patients with nervous system tumors. Second, the FAERS is a spontaneous reporting system that relies on voluntary reporting by healthcare professionals, patients, and manufacturers. As a result, the data may be subject to underreporting, reporting bias, and incomplete information, which could impact the identification and characterization of irAEs. Additionally, the lack of detailed clinical information in the FAERS database limits the ability to establish causal relationships between the reported adverse events and the use of immune checkpoint inhibitors [69]. Third, the disproportionality analysis used in this study, while widely accepted in pharmacovigilance research, may have limitations in detecting rare or delayed adverse events. The signal detection criteria applied in our analysis may have influenced the number and types of irAEs identified. Future research should consider employing additional analytical methods and exploring alternative signal detection criteria to capture a broader range of potential irAEs. Fourth, in addition to the irAEs identified in our study, it is important to acknowledge the occurrence of neuromuscular irAEs, a rare but extremely serious group of toxicities associated with ICIs, such as myocarditis and myositis, can lead to significant morbidity and mortality in cancer patients treated with ICIs [70]. Although our strict signal detection criteria did not capture these events among the NSC-irAEs, clinicians should remain vigilant for signs and symptoms of neuromuscular dysfunction in patients receiving ICIs. Early recognition and prompt management, including discontinuation of ICIs and initiation of immunosuppressive therapy, are crucial in mitigating the severity and impact of these toxicities. Further research is needed to elucidate the underlying mechanisms and risk factors associated with neuromuscular irAEs in the context of nervous system cancers.

Finally, Future research should consider incorporating additional data sources, such as other pharmacovigilance databases (e.g., EudraVigilance, VigiBase) and published literature, to identify a broader range of irAEs in patients with nervous system tumors. By integrating data from multiple sources, we aim to provide a more comprehensive analysis of the toxicity profiles of immune checkpoint inhibitors in this patient population. Despite these limitations, our study provides valuable insights into the toxicity profiles of ICIs in patients with NSCs using real-world data from the FAERS database. The findings of this study can serve as a foundation for future research aimed at better understanding and managing the side effects of immunotherapy in this patient population.

To further elucidate the mechanisms underlying irAEs in NSC patients treated with ICIs, several avenues of research should be pursued. First, prospective clinical studies with larger cohorts and longer follow-up periods are needed to validate our findings and identify additional irAEs that may not have been captured in the FAERS database. Second, preclinical models, such as genetically engineered mouse models and patient-derived xenografts, can be employed to investigate the molecular pathways involved in the development of irAEs, particularly those related to inflammatory responses, secretion of inflammatory mediators, and aberrant activation of pathological pathways. Third, the integration of multiomics data, including genomics, transcriptomics, proteomics, and metabolomics, may provide a more comprehensive understanding of the complex interplay between ICIs, the immune system, and the nervous system. Finally, the development of predictive biomarkers for irAEs in NSC patients treated with ICIs is crucial for identifying individuals at high risk and implementing personalized management strategies to mitigate the severity of these adverse events.

## Conclusions

In this study, we focused on exploring immunotherapy-related toxic effects associated with use of ICIs in NSCLC patients. Older NSC patients were more likely to experience irAEs than younger patients. irAEs in NSC patients mainly include seizure, confused state, encephalopathy, muscular weakness, gait disturbance, brain edema, hyperglycemia, urinary tract infection, atrial fibrillation, cytokine release syndrome, decreased drug tolerance, autoimmune encephalitis, granulocytopenia and COVID-19. irAEs may occur by mechanisms involving inflammatory responses, secretion of inflammatory mediators, and aberrant activation of pathological pathways (Ca2 + channels, glucocorticoid secretion and dopamine receptor signaling pathway). Therefore, a great deal of research is still needed to understand the pathways involved in secretion of inflammatory mediators, and many studies are needed to investigate and learn about irAEs and their mechanisms in NSC patients to maximize benefits.

## Supplementary Information

Below is the link to the electronic supplementary material.

Supplementary file1 (PDF 1975 KB)

Supplementary file2 (PDF 1777 KB)

Supplementary file3 (PDF 29 KB)

Supplementary file4 (PDF 58 KB)

Supplementary file5 (PDF 33 KB)

Supplementary file6 (PDF 28 KB)

Supplementary file7 (PDF 62 KB)

Supplementary file8 (PDF 30 KB)

Supplementary file9 (PDF 34 KB)

## Data Availability

All the data generated or analyzed during this study are included in this article and its supplementary files.
