# Supplementary figures and images for: Toxicity profiles of immune checkpoint inhibitors in nervous system cancer: a comprehensive disproportionality analysis using FDA adverse event reporting system
Source: Clin Exp Med. 2024 Sep 9;24(1):216. doi: 10.1007/s10238-024-01403-2 (PMC11383843; doi:10.1007/s10238-024-01403-2)

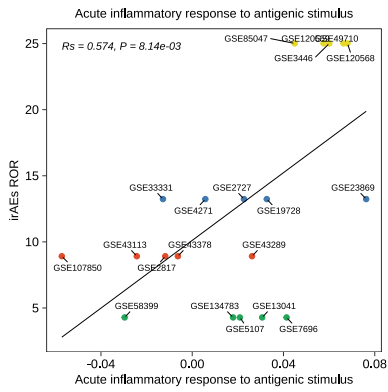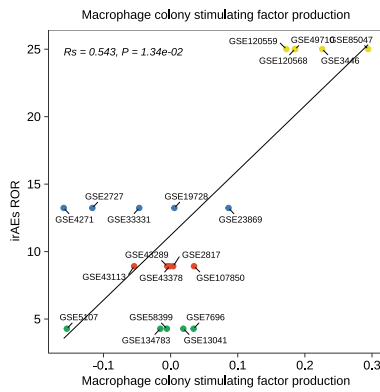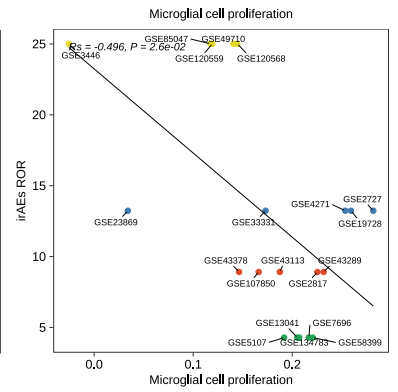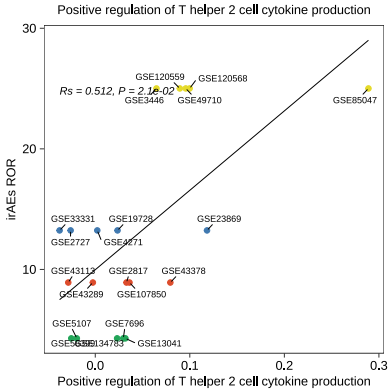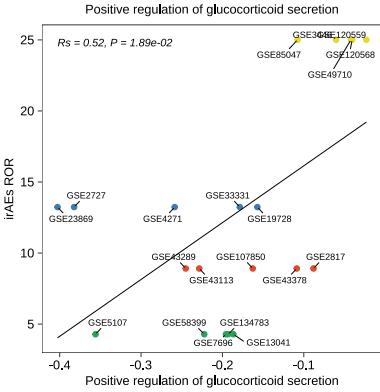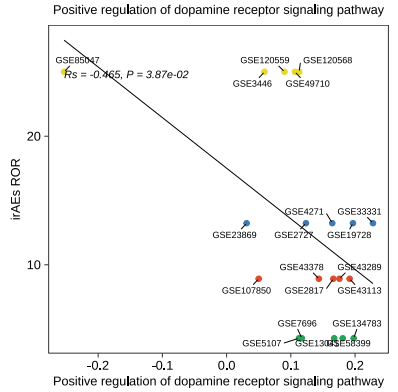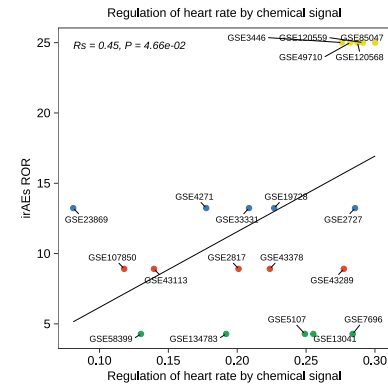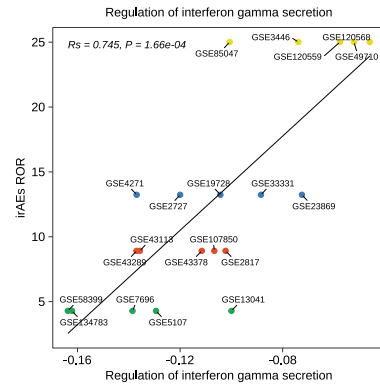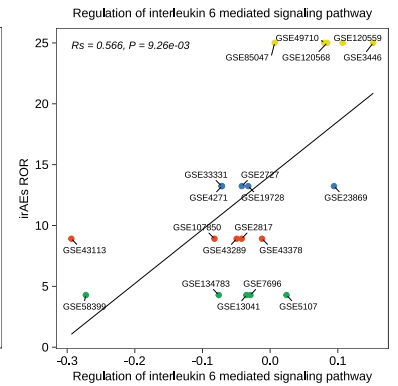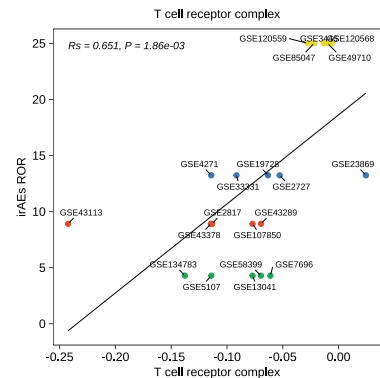

Supplement: Supplementary file 1 — Supplementary file1 (PDF 1975 KB) [file 10238_2024_1403_MOESM1_ESM.pdf]
