# Supplementary material for: Toxicity profiles of immune checkpoint inhibitors in nervous system cancer: a comprehensive disproportionality analysis using FDA adverse event reporting system
Source: Clin Exp Med. 2024 Sep 9;24(1):216. doi: 10.1007/s10238-024-01403-2 (PMC11383843; doi:10.1007/s10238-024-01403-2)
Supplement: Supplementary file 2 — Supplementary file2 (PDF 1777 KB) [file 10238_2024_1403_MOESM2_ESM.pdf]

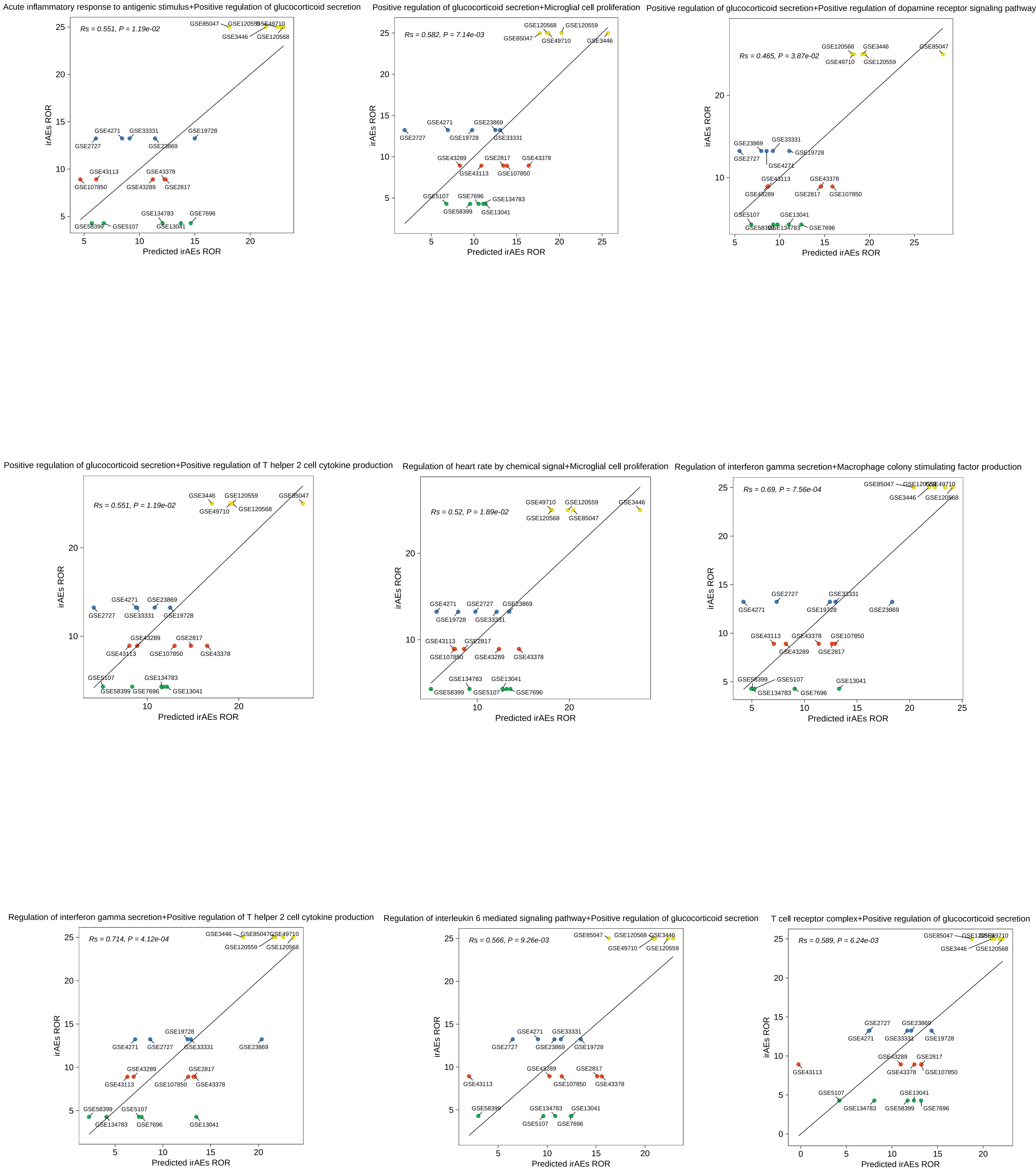
Acute inflammatory response to antigenic stimulus+Positive regulation of glucocorticoid secretion
Rs = 0.551, P = 1.19e-02
irAEs ROR
Predicted irAEs ROR
Positive regulation of glucocorticoid secretion+Microglial cell proliferation
Rs = 0.582, P = 7.14e-03
irAEs ROR
Predicted irAEs ROR
Positive regulation of glucocorticoid secretion+Positive regulation of dopamine receptor signaling pathway
Rs = 0.465, P = 3.87e-02
irAEs ROR
Predicted irAEs ROR
Positive regulation of glucocorticoid secretion+Positive regulation of T helper 2 cell cytokine production
Rs = 0.551, P = 1.19e-02
irAEs ROR
Predicted irAEs ROR
Regulation of heart rate by chemical signal+Microglial cell proliferation
Rs = 0.52, P = 1.89e-02
irAEs ROR
Predicted irAEs ROR
Regulation of interferon gamma secretion+Macrophage colony stimulating factor production
Rs = 0.69, P = 7.56e-04
irAEs ROR
Predicted irAEs ROR
Regulation of interferon gamma secretion+Positive regulation of T helper 2 cell cytokine production
Rs = 0.714, P = 4.12e-04
irAEs ROR
Predicted irAEs ROR
Regulation of interleukin 6 mediated signaling pathway+Positive regulation of glucocorticoid secretion
Rs = 0.566, P = 9.26e-03
irAEs ROR
Predicted irAEs ROR
T cell receptor complex+Positive regulation of glucocorticoid secretion
Rs = 0.589, P = 6.24e-03
irAEs ROR
Predicted irAEs ROR
