# Supplementary material for: Toxicity profiles of immune checkpoint inhibitors in nervous system cancer: a comprehensive disproportionality analysis using FDA adverse event reporting system
Source: Clin Exp Med. 2024 Sep 9;24(1):216. doi: 10.1007/s10238-024-01403-2 (PMC11383843; doi:10.1007/s10238-024-01403-2)
Supplement: Supplementary file 3 — Supplementary file3 (PDF 29 KB) [file 10238_2024_1403_MOESM3_ESM.pdf]

Drug_name	Type
Epirubicin	chemotherapy
doxorubicin	chemotherapy
Adriamycin	chemotherapy
bleomycin	chemotherapy
Mitomycin	chemotherapy
Daunorubicin	chemotherapy
methotrexate	chemotherapy
Fluorouracil	chemotherapy
Pemetrexed	chemotherapy
Gemcitabine	chemotherapy
irinotecan	chemotherapy
Etoposide	chemotherapy
topotecan	chemotherapy
Teniposide	chemotherapy
bendamustine	chemotherapy
Temozolomide	chemotherapy
Oxaliplatin	chemotherapy
Cisplatin	chemotherapy
Nedaplatin	chemotherapy
carboplatin	chemotherapy
Ifosfamide	chemotherapy
Cyclophosphamide	chemotherapy
Dacarbazine	chemotherapy
Lomustine	chemotherapy
Vinblastine	chemotherapy
vinorelbine	chemotherapy
vindesine	chemotherapy
vincristine	chemotherapy
Docetaxel	chemotherapy
paclitaxel	chemotherapy
Pembrolizumab	immunotherapy
Atezolizumab	immunotherapy
Avelumab	immunotherapy
Durvalumab	immunotherapy
Nivolumab	immunotherapy
Ipilimumab	immunotherapy
Tremelimumab	immunotherapy
