# Supplementary material for: Toxicity profiles of immune checkpoint inhibitors in nervous system cancer: a comprehensive disproportionality analysis using FDA adverse event reporting system
Source: Clin Exp Med. 2024 Sep 9;24(1):216. doi: 10.1007/s10238-024-01403-2 (PMC11383843; doi:10.1007/s10238-024-01403-2)
Supplement: Supplementary file 4 — Supplementary file4 (PDF 58 KB) [file 10238_2024_1403_MOESM4_ESM.pdf]

Accession	Type	Organism	Cancer Type	Sample Number	Citation
GSE49710	Expression profiling by array	Homo sapiens	neuroblastoma	498	Wang C, Gong B, Bushel PR, Thierry-Mieg J et al. The concordance between RNA-seq and microarray data depends on chemical treatment and transcript abundance. Nat Biotechnol 2014 Sep;32(9):926-32. PMID: 25150839
GSE85047	Expression profiling by array	Homo sapiens	neuroblastoma	283	Rajbhandari P, Lopez G, Capdevila C, Salvatori B et al. Cross-Cohort Analysis Identifies a TEAD4-MYCN Positive Feedback Loop as the Core Regulatory Element of High-Risk Neuroblastoma. Cancer Discov 2018 May;8(5):582-599. PMID: 29510988

GSE3446	Expression profiling by array	Homo sapiens	neuroblastoma	117	Asgharzadeh S, Pique-Regi R, Sposto R, Wang H et al. Prognostic significance of gene expression profiles of metastatic neuroblastomas lacking MYCN gene amplification. J Natl Cancer Inst 2006 Sep 6;98(17):1193-203. PMID: 16954472
GSE12055[illegible]	Expression profiling by array	Homo sapiens	neuroblastoma	208	Ackermann S, Cartolano M, Hero B, Welte A et al. A mechanistic classification of clinical phenotypes in neuroblastoma. Science 2018 Dec 7;362(6419):1165-1170. PMID: 30523111

GSE12056[illegible]	Expression profiling by array	Homo sapiens	neuroblastoma	186	Ackermann S, Cartolano M, Hero B, Welte A et al. A mechanistic classification of clinical phenotypes in neuroblastoma. Science 2018 Dec 7;362(6419):1165-1170. PMID: 30523111
GSE4271	Expression profiling by array	Homo sapiens	astrocytoma	100	Phillips HS, Kharbanda S, Chen R, Forrest WF et al. Molecular subclasses of high-grade glioma predict prognosis, delineate a pattern of disease progression, and resemble stages in neurogenesis. Cancer Cell 2006 Mar;9(3):157-73. PMID: 16530701
GSE2727	Expression profiling by array	Homo sapiens	astrocytoma	24	https://www.ncbi.nlm.nih.gov/geo/query/acc.cgi?acc=GSE2727
GSE23869	Expression profiling by array	Homo sapiens	astrocytoma	10	https://www.ncbi.nlm.nih.gov/geo/query/acc.cgi?acc=GSE23869

GSE33331	Expression profiling by array	Homo sapiens	astrocytoma	26	Donson AM, Birks DK, Schittone SA, Kleinschmidt-DeMasters BK et al. Increased immune gene expression and immune cell infiltration in high-grade astrocytoma distinguish long-term from short-term survivors. J Immunol 2012 Aug 15;189(4):1920-7. PMID: 22802421
GSE19728	Expression profiling by array	Homo sapiens	astrocytoma	17	Liu Z, Yao Z, Li C, Lu Y et al. Gene expression profiling in human high-grade astrocytomas. Comp Funct Genomics 2011;2011:245137. PMID: 21836821

GSE13041	Expression profiling by array	Homo sapiens	glioblastoma	27	Lee Y, Scheck AC, Cloughesy TF, Lai A et al. Gene expression analysis of glioblastomas identifies the major molecular basis for the prognostic benefit of younger age. BMC Med Genomics 2008 Oct 21;1:52. PMID: 18940004
GSE58399	Expression profiling by array	Homo sapiens	glioblastoma	105	Oh YT, Cho HJ, Kim J, Lee JH et al. Translational validation of personalized treatment strategy based on genetic characteristics of glioblastoma. PLoS One 2014;9(8):e103327. PMID: 25084005

GSE7696	Expression profiling by array	Homo sapiens	glioblastoma	80	Murat A, Migliavacca E, Gorlia T, Lambiv WL et al. Stem cell-related "self-renewal" signature and high epidermal growth factor receptor expression associated with resistance to concomitant chemoradiotherapy in glioblastoma. J Clin Oncol 2008 Jun 20;26(18):3015-24. PMID: 18565887
GSE5107	Expression profiling by array	Homo sapiens	glioblastoma	83	Tso CL, Shintaku P, Chen J, Liu Q et al. Primary glioblastomas express mesenchymal stem-like properties. Mol Cancer Res 2006 Sep;4(9):607-19. PMID: 16966431

GSE13478[illegible]	Expression profiling by array	Homo sapiens	glioblastoma	71	Berendsen S, van Bodegraven E, Seute T, Spliet WGM et al. Adverse prognosis of glioblastoma contacting the subventricular zone: Biological correlates. PLoS One 2019;14(10):e0222717. PMID: 31603915
GSE10785[illegible]	Expression profiling by array	Homo sapiens	glioma	195	Gao Y, Weenink B, van den Bent MJ, Erdem-Eraslan L et al. Expression-based intrinsic glioma subtypes are prognostic in low-grade gliomas of the EORTC22033-26033 clinical trial. Eur J Cancer 2018 May;94:168-178. PMID: 29571083

GSE43378	Expression profiling by array	Homo sapiens	glioma	50	Kawaguchi A, Yajima N, Tsuchiya N, Homma J et al. Gene expression signature-based prognostic risk score in patients with glioblastoma. Cancer Sci 2013 Sep;104(9):1205-10. PMID: 23745793
GSE43289	Expression profiling by array	Homo sapiens	glioma	40	Vital AL, Tabernero MD, Castrillo A, Rebelo O et al. Gene expression profiles of human glioblastomas are associated with both tumor cytogenetics and histopathology. Neuro Oncol 2010 Sep;12(9):991-1003. PMID: 20484145

GSE2817	Expression profiling by array	Homo sapiens	glioma	30	Turkheimer FE, Roncaroli F, Hennuy B, Herens C et al. Chromosomal patterns of gene expression from microarray data: methodology, validation and clinical relevance in gliomas. BMC Bioinformatics 2006 Dec 1;7:526. PMID: 17140431

GSE43113	Expression profiling by array	Homo sapiens	glioma	28	Erdem-Eraslan L, Gravendeel LA, de Rooi J, Eilers PH et al. Intrinsic molecular subtypes of glioma are prognostic and predict benefit from adjuvant procarbazine, lomustine, and vincristine chemotherapy in combination with other prognostic factors in anaplastic oligodendroglial brain tumors: a report from EORTC study 26951. J Clin Oncol 2013 Jan 20;31(3):328-36. PMID: 23269986
