# Supplementary material for: Toxicity profiles of immune checkpoint inhibitors in nervous system cancer: a comprehensive disproportionality analysis using FDA adverse event reporting system
Source: Clin Exp Med. 2024 Sep 9;24(1):216. doi: 10.1007/s10238-024-01403-2 (PMC11383843; doi:10.1007/s10238-024-01403-2)
Supplement: Supplementary file 5 — Supplementary file5 (PDF 33 KB) [file 10238_2024_1403_MOESM5_ESM.pdf]

irAEs	ROR	ROR025	a	IC025	PRR	yate_X2
atrial fibrillation	160.5714286	17.8680032	4	1.08550987	157.4879227	94.42927922
brain oedema	4.353461538	1.971477796	7	0.41333492	4.240059457	12.90189655
confusional state	4.703962704	2.326033011	9	0.700272299	4.542920847	19.35830065
covid-19	11.97058824	3.269992013	3	0.020769346	11.8115942	15.12931134
cytokine release s	119.8382353	12.41258588	3	0.477172062	118.115942	59.68657504
drug tolerance dec	119.8382353	12.41258588	3	0.477172062	118.115942	59.68657504
encephalitis autoir	39.93627451	8.012170384	3	0.362548507	39.37198068	38.17507725
encephalopathy	8.150753769	3.766335226	8	1.118745981	7.874396135	34.5491531
gait disturbance	3.486604938	1.59065002	7	0.182440655	3.402516849	8.873796272
granulocytopenia	19.96078431	4.957587976	3	0.206067652	19.68599034	23.87548777
hyperglycaemia	8.746233319	3.291961787	5	0.641992436	8.559126234	21.49376743
muscular weaknes	4.799745763	2.165369419	7	0.510429062	4.671251945	14.96491664
seizure	3.94727024	2.441064311	20	0.947033463	3.66250983	33.91637584
urinary tract infect	4.666705043	1.829251667	5	0.141130842	4.578137288	9.509692307
