# Supplementary material for: Toxicity profiles of immune checkpoint inhibitors in nervous system cancer: a comprehensive disproportionality analysis using FDA adverse event reporting system
Source: Clin Exp Med. 2024 Sep 9;24(1):216. doi: 10.1007/s10238-024-01403-2 (PMC11383843; doi:10.1007/s10238-024-01403-2)
Supplement: Supplementary file 6 — Supplementary file6 (PDF 28 KB) [file 10238_2024_1403_MOESM6_ESM.pdf]

reaction	ROR	ROR025	ROR975	pvalue	cancer	a
irAEs	8.914285714	3.215563045	24.71246518	0.00033311	glioma	6
irAEs	25.00952381	6.482165833	96.49186665	0.00012637	neuroblastoma	4
irAEs	4.285075072	2.974899888	6.172264299	1.64E-16	glioblastoma	51
irAEs	13.23529412	0.809972145	216.2704131	0.138984345	astrocytoma	1
