# Supplementary material for: Toxicity profiles of immune checkpoint inhibitors in nervous system cancer: a comprehensive disproportionality analysis using FDA adverse event reporting system
Source: Clin Exp Med. 2024 Sep 9;24(1):216. doi: 10.1007/s10238-024-01403-2 (PMC11383843; doi:10.1007/s10238-024-01403-2)
Supplement: Supplementary file 7 — Supplementary file7 (PDF 62 KB) [file 10238_2024_1403_MOESM7_ESM.pdf]

Factor1	Factor2	combination	Rs	P	FDR	loglikelihood_ratio_test_compared_with_F1	loglikelihood_ratio_test_compared_with_F2
Regulation of interferon gamma secretion	T cell receptor complex	Regulation of interferon gamma secretion+T cell receptor complex	0.7523	0.0001301	0.000130102	0.413858663	0.001981994
Regulation of interferon gamma secretion	Acute inflammatory response to antigenic stimulus	Regulation of interferon gamma secretion+Acute inflammatory response to antigenic stimulus	0.744544	0.0001664	0.000166442	0.362353864	0.002563344
Regulation of interferon gamma secretion	Regulation of interleukin 6 mediated signaling pathway	Regulation of interferon gamma secretion+Regulation of interleukin 6 mediated signaling pathway	0.767811	7.73E-05	7.73E-05	0.814370976	0.008716075
Regulation of interferon gamma secretion	Macrophage colony stimulating factor production	Regulation of interferon gamma secretion+Macrophage colony stimulating factor production	0.690254	0.0007559	0.000755851	0.003228206	0.008696743

Regulation of interferon gamma secretion	Calcium channel complex	Regulation of interferon gamma secretion+Calcium channel complex	0.744544	0.0001664	0.000166442	0.146995833	0.000143639
Regulation of interferon gamma secretion	Positive regulation of glucocorticoid secretion	Regulation of interferon gamma secretion+Positive regulation of glucocorticoid secretion	0.760055	0.0001008	0.000100791	0.136247073	0.001398512
Regulation of interferon gamma secretion	Positive regulation of T helper 2 cell cytokine production	Regulation of interferon gamma secretion+Positive regulation of T helper 2 cell cytokine production	0.713521	0.0004118	0.00041178	0.032583815	0.001565496
Regulation of interferon gamma secretion	Regulation of heart rate by chemical signal	Regulation of interferon gamma secretion+Regulation of heart rate by chemical signal	0.775567	5.88E-05	5.88E-05	0.086097207	0.000166321
Regulation of interferon gamma secretion	Microglial cell proliferation	Regulation of interferon gamma secretion+Microglial cell proliferation	0.736788	0.0002111	0.000211149	0.280776866	0.004077061

Regulation of interferon gamma secretion	Positive regulation of dopamine receptor signaling pathway	Regulation of interferon gamma secretion+Positive regulation of dopamine receptor signaling pathway	0.744544	0.0001664	0.000166442	0.020940733	0.000135072
T cell receptor complex	Acute inflammatory response to antigenic stimulus	T cell receptor complex+Acute inflammatory response to antigenic stimulus	0.612698	0.004078	0.004077972	0.195920567	0.308012534
T cell receptor complex	Regulation of interleukin 6 mediated signaling pathway	T cell receptor complex+Regulation of interleukin 6 mediated signaling pathway	0.628209	0.0030156	0.003015571	0.118963239	0.549930996
T cell receptor complex	Macrophage colony stimulating factor production	T cell receptor complex+Macrophage colony stimulating factor production	0.527385	0.0168657	0.016865725	0.000978394	0.671502385
T cell receptor complex	Calcium channel complex	T cell receptor complex+Calcium channel complex	0.651476	0.0018596	0.001859595	0.658458024	0.056149738

T cell receptor complex	Positive regulation of glucocorticoid secretion	T cell receptor complex+Positive regulation of glucocorticoid secretion	0.589431	0.0062381	0.006238085	0.008158204	0.013611243
T cell receptor complex	Positive regulation of T helper 2 cell cytokine production	T cell receptor complex+Positive regulation of T helper 2 cell cytokine production	0.604942	0.0047154	0.004715414	0.031396111	0.2804105
T cell receptor complex	Regulation of heart rate by chemical signal	T cell receptor complex+Regulation of heart rate by chemical signal	0.64372	0.0021942	0.002194218	0.162789157	0.03852698
T cell receptor complex	Microglial cell proliferation	T cell receptor complex+Microglial cell proliferation	0.566164	0.00926	0.00925998	0.045536746	0.139266412
T cell receptor complex	Positive regulation of dopamine receptor signaling pathway	T cell receptor complex+Positive regulation of dopamine receptor signaling pathway	0.604942	0.0047154	0.004715414	0.130825591	0.105380431

Acute inflammatory response to antigenic stimulus	Regulation of interleukin 6 mediated signaling pathway	Acute inflammatory response to antigenic stimulus+Regulation of interleukin 6 mediated signaling pathway	0.550652	0.0118683	0.01186827	0.174247593	0.524065516
Acute inflammatory response to antigenic stimulus	Macrophage colony stimulating factor production	Acute inflammatory response to antigenic stimulus+Macrophage colony stimulating factor production	0.51963	0.0188632	0.018863169	0.000791173	0.271925032
Acute inflammatory response to antigenic stimulus	Calcium channel complex	Acute inflammatory response to antigenic stimulus+Calcium channel complex	0.542897	0.0133791	0.013379117	0.119174489	0.010704707
Acute inflammatory response to antigenic stimulus	Positive regulation of glucocorticoid secretion	Acute inflammatory response to antigenic stimulus+Positive regulation of glucocorticoid secretion	0.550652	0.0118683	0.01186827	0.004318624	0.00503297

Acute inflammatory response to antigenic stimulus	Positive regulation of T helper 2 cell cytokine production	Acute inflammatory response to antigenic stimulus+Positive regulation of T helper 2 cell cytokine production	0.527385	0.0168657	0.016865725	0.028792796	0.162885239
Acute inflammatory response to antigenic stimulus	Regulation of heart rate by chemical signal	Acute inflammatory response to antigenic stimulus+Regulation of heart rate by chemical signal	0.589431	0.0062381	0.006238085	0.474236664	0.062155515
Acute inflammatory response to antigenic stimulus	Microglial cell proliferation	Acute inflammatory response to antigenic stimulus+Microglial cell proliferation	0.473096	0.0351381	0.035138069	0.048698676	0.099942949
Acute inflammatory response to antigenic stimulus	Positive regulation of dopamine receptor signaling pathway	Acute inflammatory response to antigenic stimulus+Positive regulation of dopamine receptor signaling pathway	0.550652	0.0118683	0.01186827	0.07632928	0.042524335

Regulation of interleukin 6 mediated signaling pathway	Macrophage colony stimulating factor production	Regulation of interleukin 6 mediated signaling pathway+Macrophage colony stimulating factor production	0.542897	0.0133791	0.013379117	0.000908356	0.122040088
Regulation of interleukin 6 mediated signaling pathway	Calcium channel complex	Regulation of interleukin 6 mediated signaling pathway+Calcium channel complex	0.581675	0.0071387	0.007138686	0.345985202	0.011323604
Regulation of interleukin 6 mediated signaling pathway	Positive regulation of glucocorticoid secretion	Regulation of interleukin 6 mediated signaling pathway+Positive regulation of glucocorticoid secretion	0.566164	0.00926	0.00925998	0.015776454	0.008194058
Regulation of interleukin 6 mediated signaling pathway	Positive regulation of T helper 2 cell cytokine production	Regulation of interleukin 6 mediated signaling pathway+Positive regulation of T helper 2 cell cytokine production	0.504118	0.023423	0.023423028	0.028980847	0.066146203

Regulation of interleukin 6 mediated signaling pathway	Regulation of heart rate by chemical signal	Regulation of interleukin 6 mediated signaling pathway+Regulation of heart rate by chemical signal	0.573919	0.0081431	0.008143076	0.472664521	0.026506112
Regulation of interleukin 6 mediated signaling pathway	Microglial cell proliferation	Regulation of interleukin 6 mediated signaling pathway+Microglial cell proliferation	0.51963	0.0188632	0.018863169	0.107910171	0.091633675
Regulation of interleukin 6 mediated signaling pathway	Positive regulation of dopamine receptor signaling pathway	Regulation of interleukin 6 mediated signaling pathway+Positive regulation of dopamine receptor signaling pathway	0.542897	0.0133791	0.013379117	0.104854878	0.02472468
Macrophage colony stimulating factor production	Calcium channel complex	Macrophage colony stimulating factor production+Calcium channel complex	0.511874	0.0210451	0.021045054	0.322082607	0.000100844

Macrophage colony stimulating factor production	Positive regulation of glucocorticoid secretion	Macrophage colony stimulating factor production+Positive regulation of glucocorticoid secretion	0.542897	0.0133791	0.013379117	0.750824764	0.00167224
Macrophage colony stimulating factor production	Positive regulation of T helper 2 cell cytokine production	Macrophage colony stimulating factor production+Positive regulation of T helper 2 cell cytokine production	0.542897	0.0133791	0.013379117	0.743964372	0.006785973
Macrophage colony stimulating factor production	Regulation of heart rate by chemical signal	Macrophage colony stimulating factor production+Regulation of heart rate by chemical signal	0.511874	0.0210451	0.021045054	0.179590442	0.000118108
Macrophage colony stimulating factor production	Microglial cell proliferation	Macrophage colony stimulating factor production+Microglial cell proliferation	0.51963	0.0188632	0.018863169	0.612716283	0.002512046

Macrophage colony stimulating factor production	Positive regulation of dopamine receptor signaling pathway	Macrophage colony stimulating factor production+Positive regulation of dopamine receptor signaling pathway	0.542897	0.0133791	0.013379117	0.841004648	0.000878354
Calcium channel complex	Positive regulation of glucocorticoid secretion	Calcium channel complex+Positive regulation of glucocorticoid secretion	0.480851	0.031854	0.031854032	0.019774082	0.301174066
Calcium channel complex	Positive regulation of T helper 2 cell cytokine production	Calcium channel complex+Positive regulation of T helper 2 cell cytokine production	0.488607	0.0288152	0.028815219	0.00510849	0.336859758
Calcium channel complex	Regulation of heart rate by chemical signal	Calcium channel complex+Regulation of heart rate by chemical signal	0.51963	0.0188632	0.018863169	0.07016827	0.141612278
Calcium channel complex	Microglial cell proliferation	Calcium channel complex+Microglial cell proliferation	0.418806	0.0660768	0.06607683	0.012364599	0.319007058

Calcium channel complex	Positive regulation of dopamine receptor signaling pathway	Calcium channel complex+Positive regulation of dopamine receptor signaling pathway	0.418806	0.0660768	0.06607683	0.062444336	0.549327797
Positive regulation of glucocorticoid secretion	Positive regulation of T helper 2 cell cytokine production	Positive regulation of glucocorticoid secretion+Positive regulation of T helper 2 cell cytokine production	0.550652	0.0118683	0.01186827	0.010576347	0.046054269
Positive regulation of glucocorticoid secretion	Regulation of heart rate by chemical signal	Positive regulation of glucocorticoid secretion+Regulation of heart rate by chemical signal	0.527385	0.0168657	0.016865725	0.273926404	0.035085851
Positive regulation of glucocorticoid secretion	Microglial cell proliferation	Positive regulation of glucocorticoid secretion+Microglial cell proliferation	0.581675	0.0071387	0.007138686	0.011510418	0.019205649
Positive regulation of glucocorticoid secretion	Positive regulation of dopamine receptor signaling pathway	Positive regulation of glucocorticoid secretion+Positive regulation of dopamine receptor signaling pathway	0.46534	0.0386801	0.038680102	0.018238437	0.008998517

Positive regulation of T helper 2 cell cytokine production	Regulation of heart rate by chemical signal	Positive regulation of T helper 2 cell cytokine production+Regulation of heart rate by chemical signal	0.550652	0.0118683	0.01186827	0.147080037	0.004938484
Positive regulation of T helper 2 cell cytokine production	Microglial cell proliferation	Positive regulation of T helper 2 cell cytokine production+Microglial cell proliferation	0.46534	0.0386801	0.038680102	0.091967803	0.034032759
Positive regulation of T helper 2 cell cytokine production	Positive regulation of dopamine receptor signaling pathway	Positive regulation of T helper 2 cell cytokine production+Positive regulation of dopamine receptor signaling pathway	0.511874	0.0210451	0.021045054	0.392143437	0.033153313
Regulation of heart rate by chemical signal	Microglial cell proliferation	Regulation of heart rate by chemical signal+Microglial cell proliferation	0.51963	0.0188632	0.018863169	0.000910704	0.008840533

Regulation of heart rate by chemical signal	Positive regulation of dopamine receptor signaling pathway	Regulation of heart rate by chemical signal+Positive regulation of dopamine receptor signaling pathway	0.51963	0.0188632	0.018863169	0.035919622	0.120799577
Microglial cell proliferation	Positive regulation of dopamine receptor signaling pathway	Microglial cell proliferation+Positive regulation of dopamine receptor signaling pathway	0.41105	0.0717884	0.0717884	0.178668521	0.046572835
