# Supplementary material for: Toxicity profiles of immune checkpoint inhibitors in nervous system cancer: a comprehensive disproportionality analysis using FDA adverse event reporting system
Source: Clin Exp Med. 2024 Sep 9;24(1):216. doi: 10.1007/s10238-024-01403-2 (PMC11383843; doi:10.1007/s10238-024-01403-2)
Supplement: Supplementary file 8 — Supplementary file8 (PDF 30 KB) [file 10238_2024_1403_MOESM8_ESM.pdf]

Gene	Rs	P
AHR	0.302471026	0.194903648
B2M	0.341249363	0.140893632
BACH2	0.620453387	0.003513573
BANK1	0.255937022	0.276085611
BTN3A1	0.217158686	0.357743865
BTN3A2	0.224914353	0.340400731
CCL5	0.49636271	0.026009021
CD40	0.186136016	0.432025769
CD58	0.23267002	0.323559981
CD79A	0.418806037	0.06607683
CD79B	0.434317371	0.055687329
CLEC5A	-0.418806037	0.06607683
CX3CL1	0.426561704	0.060714089
CXCL10	0.046534004	0.845540608
CYBB	0.620453387	0.003513573
EDNRB	-0.155113347	0.513761221
ENTPD1	-0.193891684	0.412732981
EVI2B	0.34900503	0.1315062
FOXP3	0.124090677	0.602199926
FUCA1	0.465340041	0.038680102
GPNMB	0.434317371	0.055687329
GZMA	NA	0.097729165
HLA-DPA1	0.217158686	0.357743865
HLA-DPB1	0.442073039	0.050983592
HS3ST2	0.255937022	0.276085611
HSD17B11	0.573919383	0.008143076
ICAM1	0.387783367	0.091141863
IFIT2	0.333493696	0.150741897
IFIT3	0.007755667	0.974112086
IL10	0.49636271	0.026009021
IL12A	0.170624682	0.472002597
IL1A	-0.286959692	0.219937738
IL1B	0.403294702	0.077861483
IL2RA	0.449828706	0.046589845
IL32	NA	0.197008217
IRF7	0.550652381	0.01186827
ISG20	0.434317371	0.055687329
ITGB2	NA	0.110083807
LAIR1	0.387783367	0.091141863
LGMN	0.317982361	0.17185438
LILRA4	0.34900503	0.1315062
MX2	0.403294702	0.077861483
