# Supplementary material for: Toxicity profiles of immune checkpoint inhibitors in nervous system cancer: a comprehensive disproportionality analysis using FDA adverse event reporting system
Source: Clin Exp Med. 2024 Sep 9;24(1):216. doi: 10.1007/s10238-024-01403-2 (PMC11383843; doi:10.1007/s10238-024-01403-2)
Supplement: Supplementary file 9 — Supplementary file9 (PDF 34 KB) [file 10238_2024_1403_MOESM9_ESM.pdf]

| ID | Description | GeneRatio | BgRatio | pvalue | qvalue | geneID |
|---|---|---|---|---|---|---|
| GO:0042098 | T cell proliferation | 3/8 | 195/18862 | 5.86E-05 | 0.001171025 | 6352/3586/3559 |
| GO:0032607 | interferon-alpha production | 2/8 | 28/18862 | 5.92E-05 | 0.001171025 | 3586/3665 |
| GO:0050670 | regulation of lymphocyte proliferation | 3/8 | 221/18862 | 8.51E-05 | 0.001441239 | 6352/3586/3559 |
| GO:0046427 | positive regulation of receptor signaling pathway via JAK-STAT | 2/8 | 44/18862 | 0.00014759 | 0.001727138 | 6352/3586 |
| GO:0050863 | regulation of T cell activation | 3/8 | 327/18862 | 0.000271037 | 0.00220392 | 6352/3586/3559 |
| GO:0002437 | inflammatory response to antigenic stimulus | 2/8 | 62/18862 | 0.000293899 | 0.002236963 | 3586/3559 |
| GO:0050727 | regulation of inflammatory response | 3/8 | 366/18862 | 0.000377444 | 0.00235296 | 6352/3586/3559 |
| GO:0071346 | cellular response to interferon-gamma | 2/8 | 177/18862 | 0.002362394 | 0.006404183 | 6352/3665 |
| GO:0001819 | positive regulation of cytokine production | 2/8 | 437/18862 | 0.013670683 | 0.012077483 | 3586/3665 |
| GO:0035722 | interleukin-12-mediated signaling pathway | 1/8 | 47/18862 | 0.019764908 | 0.014380041 | 3586 |
| GO:0032677 | regulation of interleukin-8 production | 1/8 | 97/18862 | 0.040415349 | 0.020299454 | 3586 |
| GO:0032652 | regulation of interleukin-1 production | 1/8 | 119/18862 | 0.049380272 | 0.023211629 | 3586 |
